# Chronic Brucella infection associated with amyloid light chain cardiac amyloidosis: a case report

**DOI:** 10.3389/fcvm.2026.1703993

**Published:** 2026-03-12

**Authors:** RuiXia He, HaiJun Wang, LingMin Wu, Li Li, XinYuan Xu, GuoLi Ma, BaYaEr Qi, LiNa Ji, XiaoPing Liu

**Affiliations:** 1 Department of Cardiology, Ordos Clinical College of Inner Mongolia Medical University, Ordos Central Hospital, Ordos, Inner Mongolia Autonomous Region, China; 2 Clinical EP Laboratory and Arrhythmia Center, Fuwai Hospital and Cardiovascular Institute, Chinese Academy of Medical Sciences & Peking Union Medical College, Beijing, China; 3 Clinical Pathology, Fuwai Hospital and Cardiovascular Institute, Chinese Academy of Medical Sciences & Peking Union Medical College, Beijing, China

**Keywords:** amyloid light chain, Brucella infections, cardiac amyloidosis, immunity, inflammation

## Abstract

**Background:**

Amyloidosis is a rare group of diseases characterized by extracellular deposition of amyloid fibrils. The most common types are amyloid light chain (AL) and transthyretin (ATTR), while serum amyloid A (AA) type is relatively rare. Infections are often associated with AA amyloidosis, whereas AL amyloidosis typically results from abnormal proliferation of monoclonal plasma cells. However, the relationship between AL amyloidosis and infections remains unclear.

**Case presentation:**

We present the case of a 69-year-old male patient with chronic brucellosis complicated by heart failure. Laboratory tests showed significantly elevated myocardial enzymes and N-terminal pro-B type natriuretic peptide. Cardiac echocardiography and cardiac magnetic resonance imaging revealed abnormal myocardial hypertrophy, and the patient showed poor response to myocarditis-targeted treatment.

**Outcome:**

Genetic testing for inherited cardiomyopathies ruled out Fabry disease, hereditary hypertrophic cardiomyopathy, and ATTR amyloidosis-associated abnormal genes. Serum immunofixation electrophoresis indicated positivity for lambda light chain. AL cardiac amyloidosis was confirmed by myocardial biopsy.

**Conclusion:**

We report a case of chronic Brucella infection and AL-type cardiac amyloidosis, with a high overlap in clinical onset time. However, additional studies are needed to confirm the potential relationship between Brucella infection and AL-type cardiac amyloidosis.

## 1 Introduction

Amyloidosis is a rare group of diseases characterized by abnormal extracellular deposition of amyloid fibrils. Biopsy of affected organs remains the gold standard for diagnosing amyloidosis (1). The organs most commonly affected by amyloidosis include the heart and kidney. Cardiac amyloidosis has an estimated incidence of 18–55 per 100,000 person-years (2). However, the actual incidence may be higher due to underdiagnosis, especially in elderly populations.

To date, approximately 60 types of amyloid proteins have been identified, with amyloid light chain (AL) and transthyretin (ATTR) being the most common types, The detection rate of wild-type ATTR has increased significantly in recent years, while the serum amyloid A (AA) type is relatively rare (3). AL is derived from monoclonal plasma cells in the bone marrow and is produced by immunoglobulin (Ig) light chains composed of kappa or lambda proteins, also known as primary systemic amyloidosis. In contrast, AA amyloidosis is caused by sustained excessive production of serum amyloid A (SAA) protein due to chronic inflammation.

Although numerous studies on amyloidosis have been conducted, only a few have reported correlations between chronic infections and AL amyloidosis. Here, we present an unusual case of cardiac amyloidosis with significant overlap with a period of Brucella infection. The clinical manifestations suggest that chronic Brucella infection may be involved in the pathological process of cardiac amyloidosis. Despite the lack of direct evidence, the present case provides a biological hypothesis for the association of cardiac amyloidosis and Brucella infection.

## 2 Case presentation

**Figure 1:**
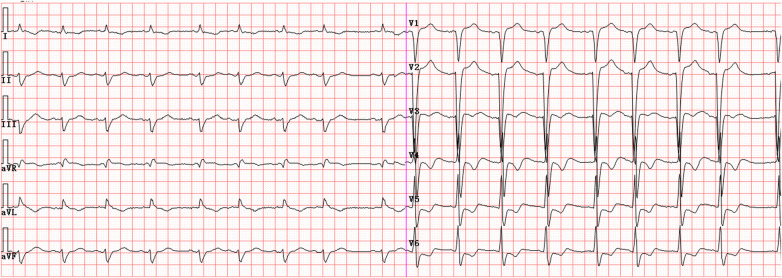
Electrocardiogram on admission showed sinus rhythm, pathological Q waves in leads V1–V3, and non-specific intraventricular conduction block.

**Figure 2:**
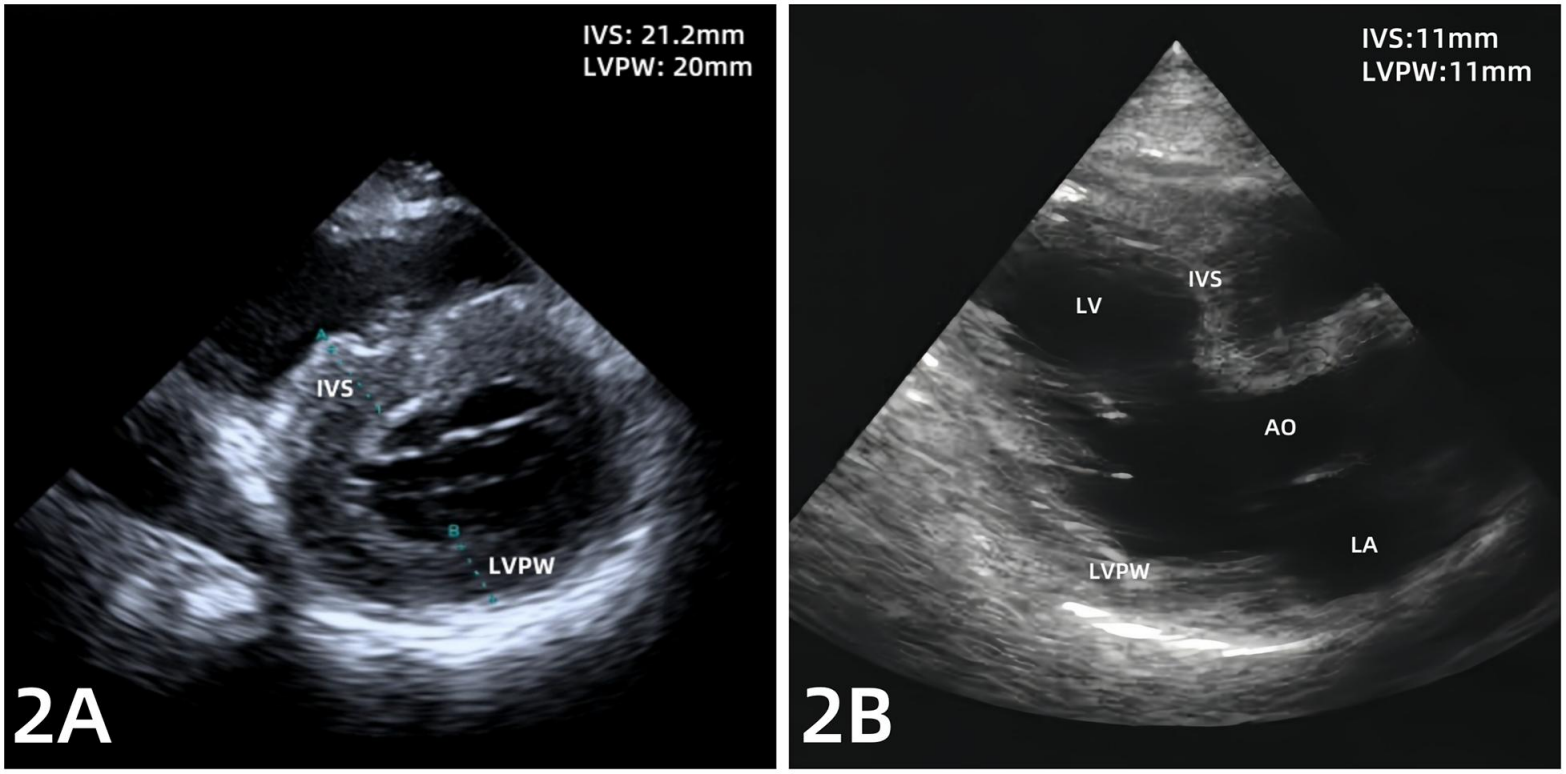
**(A)** Echocardiography on admission showed interventricular septum (IVS) of 21.2 mm and a left ventricular posterior wall (LVPW) of 20 mm. **(B)** Non-hypertrophied myocardium on echocardiography 2 years ago.

**Figure 3:**
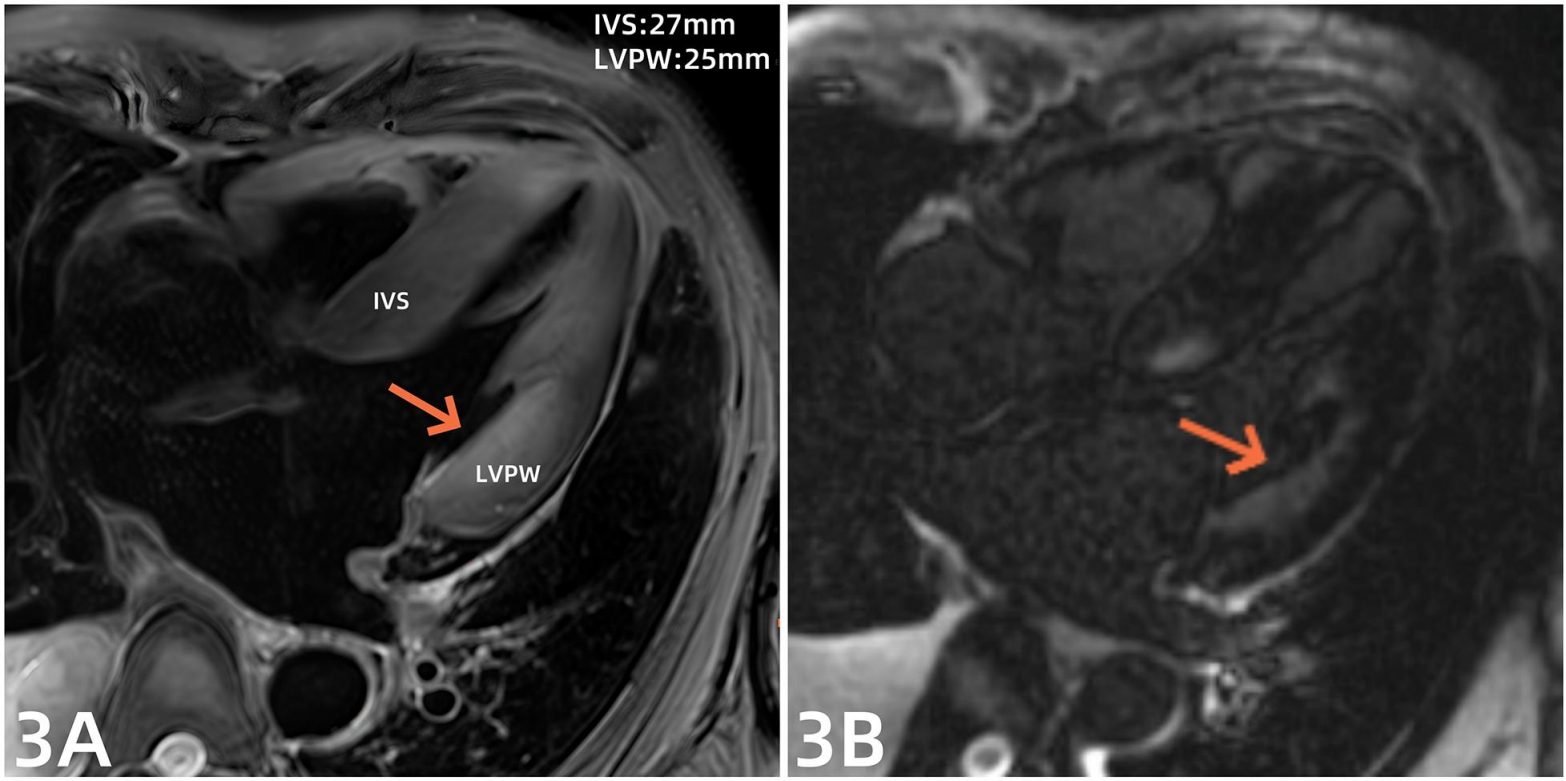
**(A)** CMR showed myocardial edema IVS 27 mm and LVPW 25 mm. **(B)** Late gadolinium enhancement imaging and accumulation of gadolinium contrast.

A 69-year-old man was well until 2019, when he presented with an acute anterior wall myocardial infarction treated with percutaneous coronary intervention (PCI). He was subsequently diagnosed with brucellosis in 2022 but received no systematic treatment. In 2023, he sustained a tibial fracture and later underwent PCI to treat unstable angina pectoris. In November 2024, he developed symptoms of dyspnea. A brucellosis tube agglutination test revealed a titer of 1:200 (normal value 1:25). He initially received oral rifapentine (0.4 g twice a week) combined with doxycycline (100 mg twice a day) at a local hospital for 2 weeks. As his dyspnea persisted, the patient was transferred to our hospital 1 week later. On admission, the patient presented with dyspnea but had no fever or chest pain. His blood pressure was 90/55 mmHg (1 mmHg = 0.133 kPa). Physical examination revealed low breath sounds of both lungs, with wet rales in the right lung. Heart sounds were low and dull, with regular rhythm. Mild edema was observed in both lower limbs. Electrocardiography showed sinus rhythm, pathological Q waves in leads V1–V3, and non-specific intraventricular conduction block (Figure 1). Laboratory test results revealed N-terminal pro-B type natriuretic peptide (NT-proBNP) level of 28,306 pg/mL (normal <125 pg/mL), high-sensitivity cardiac troponin T (hs-cTnT) range of 0.157–0.195 µg/L (normal <0.014 µg/L), serum albumin level of 30.6 g/L (normal 40–55 g/L), and albumin to globin ratio of 1.3 (normal 1.2–2.4). Echocardiography demonstrated an interventricular septum (IVS) of 21.2 mm, a left ventricular posterior wall (LVPW) of 20 mm (Figure 2A), and a left ventricular ejection fraction of 65%. Thoracic ultrasound revealed a large pleural effusion on the right side (120 mm). Cardiac magnetic resonance (CMR) imaging showed myocardial edema with IVS of 27 mm and LVPW of 25 mm (Figure 3A), as well as late gadolinium enhancement, indicating myocardial fibrosis and edema (Figure 3B). Given the lack of myocardial hypertrophy on echocardiography in this patient 2 years earlier (Figure 2B), the possibility of Brucella infection leading to fulminant myocarditis was considered. After admission, the patient developed ventricular tachycardia with loss of consciousness and experienced conversion to sinus rhythm after electrical defibrillation. Immediately after the conversion, the patient was administered the following drugs: aspirin at 100 mg once a day, rosuvastatin calcium at 10 mg once a day, torsemide at 10 mg once a day, spironolactone at 20 mg once a day, recombinant human brain natriuretic peptide at 0.0075 µg/kg/min, and sacubitril and valsartan 25 mg twice a day. The patient was also administered anti-Brucella therapy, consisting of continuous oral administration of doxycycline (100 mg twice daily) and rifampicin (900 mg once daily). In addition, the patient received Ig at 20 g once a day for 3 days, followed by 10 g per day for 5 days. Methylprednisolone was also administered at 200 mg once a day for 3 days, followed by a phased reduction. Concurrent myocardial nutritional support was provided. After 2 weeks of treatment, the dyspnea symptoms were relieved, and the patient was discharged from the hospital.

**Figure 4:**
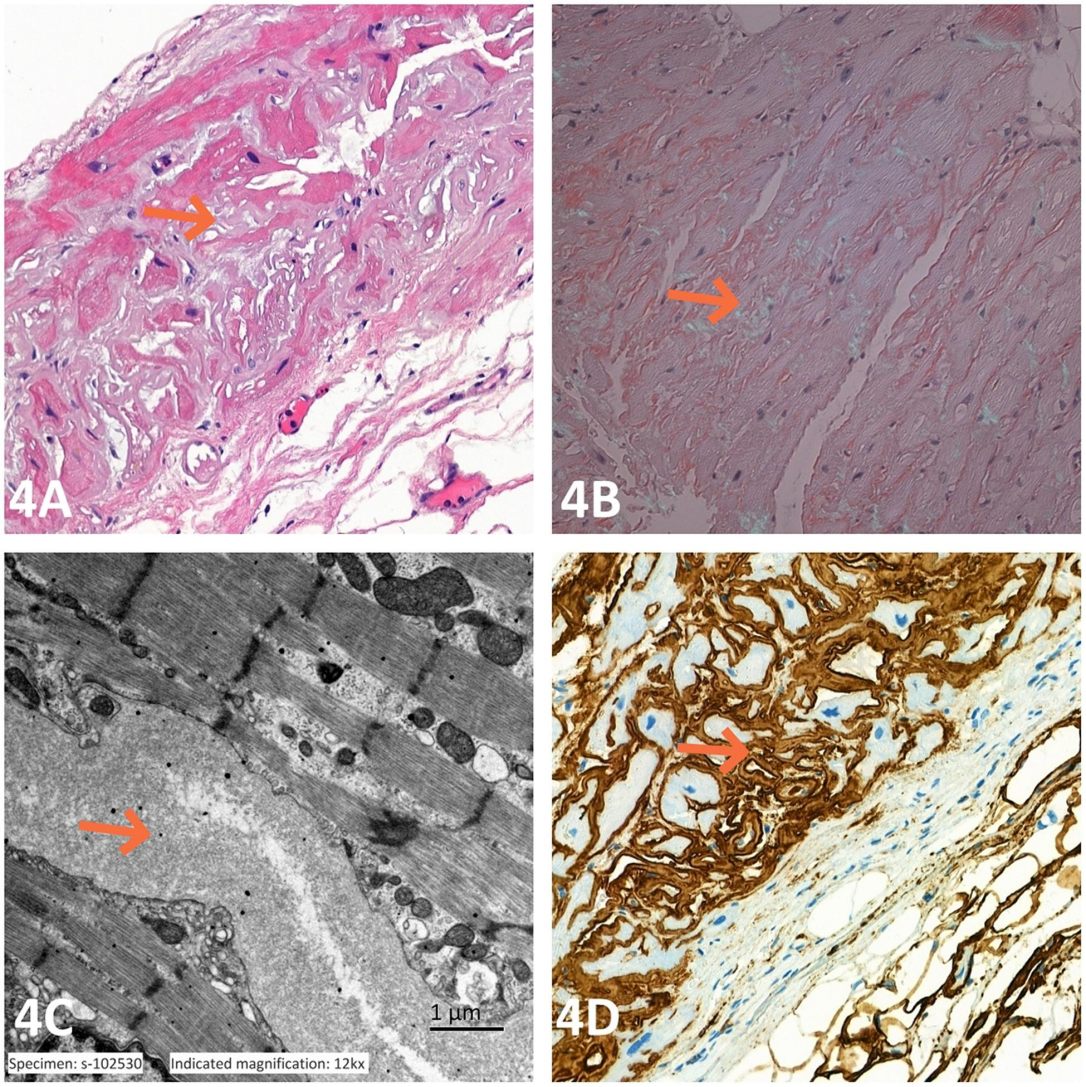
**(A)** HE staining revealed homogeneous pale pink amorphous material deposits in the myocardial interstitium. **(B)** Congo red staining and polarized light microscopy revealed an amyloid material green in color. **(C)** Transmission electron microscopy revealed disorganized and unbranched amyloid fibrils in the myocardial interstitium. **(D)** Immunohistochemical staining revealed strong positive staining for immunoglobulin light chain lambda.

One month after discharge, the patient was readmitted with dyspnea, with elevated levels of NT-proBNP (>35,000 pg/mL) and hs-cTnT (0.25 µg/L). Echocardiography revealed IVS of 22 mm and LVPW of 21 mm, consistent with previous findings. Genetic testing for inherited cardiomyopathies ruled out Fabry disease, hereditary hypertrophic cardiomyopathy, and ATTR amyloidosis-associated abnormal genes. Serum immunofixation electrophoresis indicated positivity for lambda light chain. Endomyocardial biopsy (EMB) with hematoxylin and eosin (HE) staining revealed homogeneous deposits of a light pinkish substance in the myocardial interstitium. Polarized light microscopy and Congo red staining revealed apple-green birefringence, indicating amyloid material. Transmission electron microscopy of the myocardial interstitium revealed unorganized unbranched amyloid fibers. Immunohistochemical staining indicated strong positivity for Ig light chain lambda (Figures 4A–D). On the basis of the biopsy results, the patient was diagnosed with cardiac AL amyloidosis. Bone marrow aspiration was planned to clarify the characteristics of plasma cell disease, but the patient refused further treatment and died 1 month after myocardial biopsy.

## 3 Discussion

The present case describes a patient with chronic brucellosis accompanied by abnormal myocardial hypertrophy. EMB confirmed the diagnosis of AL cardiac amyloidosis. The two conditions had a high temporal overlap in clinical onset. Although the coincidence of their coexistence cannot be ruled out, this case also provides a plausible biological hypothesis for an association between AL cardiac amyloidosis and chronic Brucella infection.

Previous studies have reported that chronic infection triggers a persistent inflammatory response (4) and leads to chronically elevated levels of SAA protein, an acute-phase response protein synthesized by the liver. SAA proteins are incompletely degraded by proteases in chronic inflammation and form insoluble amyloid fibril fragments, namely, AA proteins (5). AA protein deposition in the heart can lead to AA-type myocardial amyloidosis, leading to thickening of the ventricular wall and reduced compliance of the myocardium, resulting in diastolic dysfunction of the heart (6). In addition, chronic infections themselves may exacerbate myocardial injury through various inflammatory factors, including tumor necrosis factor alpha (TNF-α), interleukin (IL)-6, and IL-1 (7).

The underlying etiology of AL amyloidosis is clonal plasma cell expansion [also known as plasma cell dyscrasia (PCD)], producing amyloidogenic Ig light chains that aggregate to form insoluble fibrils, which are deposited in tissues and cause organ dysfunction (8). Cardiac dysfunction in AL amyloidosis may also result from direct light chain toxicity (9). With >75% of patients exhibiting symptoms of cardiac amyloid infiltration at diagnosis, the heart is the most common organ affected by AL amyloidosis (10).

While previous studies have reported no significant correlation between AL amyloidosis and chronic infections, recent reports have suggested that patients with PCD and AL amyloidosis may develop these conditions due to immune dysregulation caused by chronic infectious diseases, such as human immunodeficiency virus (HIV) and hepatitis B virus (HBV) (11). HBV causes chronic immunological stimulation, while HIV antigens act as superantigens and stimulate B-cell proliferation, thereby determining Ig production. Misfolded Igs accumulate as amyloid fibrils and lead to AL amyloidosis. HIV infection also depletes CD4+ T cells, which induces immunodeficiency (12). Moreover, some studies have reported a correlation between monoclonal proliferation of plasma cells and HIV (13). Therefore, chronic infections may increase the risk of AL amyloidosis through immune dysregulation.

Brucella is a gram-negative intracellular coccobacillus transmitted through unpasteurized dairy products or contact with infected animals (14). Its virulence factors [e.g., lipopolysaccharide (LPS) and type IV secretion system] allow it to evade host immune recognition and survive for extended periods of time in certain cells, including macrophages, resulting in chronic infections (15). Once infected, the host immune system activates proinflammatory factors (e.g., TNF-α and IL-6), triggering inflammatory response. Moreover, miR-21 expression is significantly elevated in patients in the acute phase and may be involved in myocardial injury by regulating inflammatory pathways (16). In addition, granuloma formation and complement activation further injure the normal structure of the myocardium (17). For these reasons, Brucella infection has been previously reported to cause myocarditis and endocarditis (18).

In the present case, the patient had brucellosis for 3 years without regular treatment. Compared with echocardiography findings from 2 years earlier, non-invasive cardiac imaging indicated significant thickening of the IVS and the LVPW. Serological test results and serum immunofixation electrophoresis revealed positive lambda light chains. EMB confirmed AL amyloid deposition in the myocardium. Although the possibility of coexistence of the two diseases cannot be excluded, the onset times of cardiac amyloidosis and brucellosis significantly overlapped, suggesting that chronic brucellosis may correlate with cardiac AL amyloidosis. Even though there is no direct evidence that Brucella infection causes monoclonal gammopathy, Brucella, as an intracellular pathogen, can persist in macrophages to form chronic infections. Moreover, secretory IgM plays a protective role in immunity against Brucella during infection (19). Therefore, these findings suggest that persistent chronic infections and antigenic stimulation may lead to clonal proliferation of B cells that produce monoclonal Igs, thereby leading to a dominant expansion of specific B-cell clones. Furthermore, the multisystem immune abnormalities caused by brucellosis may create favorable conditions for clonal proliferation of plasma cells. Therefore, we hypothesize that these factors may be associated with AL cardiac amyloidosis.

## 4 Conclusion

We report a case of chronic Brucella infection and AL-type cardiac amyloidosis with high overlap in clinical onset time, but additional studies are needed to verify the relationship between them.

## 5 Limitations

(1) The patient suffered an unexpected sudden death, which prevented completion of bone marrow aspiration. Consequently, plasma cell disease could not be definitively confirmed, nor could its specific characteristics be fully elucidated.
(2) This study was conducted in a region endemic for brucellosis. Therefore, the possibility of its coexistence with cardiac amyloidosis cannot be ruled out. Moreover, the association between Brucella infection and AL cardiac amyloidosis is a hypothesis. Thus, confirmation of their specific causal relationship needs further support from additional cohort studies or mechanistic research.

## Data Availability

The original contributions presented in the study are included in the article, further inquiries can be directed to the corresponding author.
